# Diarrheagenic *Escherichia coli* Carrying Supplementary Virulence Genes Are an Important Cause of Moderate to Severe Diarrhoeal Disease in Mexico

**DOI:** 10.1371/journal.pntd.0003510

**Published:** 2015-03-04

**Authors:** Sandra Patzi-Vargas, Mussaret Bano Zaidi, Iza Perez-Martinez, Magda León–Cen, Alba Michel-Ayala, Damien Chaussabel, Teresa Estrada-Garcia

**Affiliations:** 1 Department of Molecular Biomedicine, CINVESTAV-IPN, México DF, México; 2 Microbiology Research Laboratory and Paediatric Emergency Department, Hospital General O’Horán, Mérida, Yucatán, México; 3 Infectious Diseases Research Unit, Hospital Regional de Alta Especialidad de La Península de Yucatán, Mérida, Yucatán, México; 4 Department of Systems Immunology, Benaroya Research Institute, Seattle, Washington, United States of America; Institut Pasteur, FRANCE

## Abstract

Diarrheagenic *Escherichia coli* (DEC) cause acute and persistent diarrhoea worldwide, but little is known about their epidemiology in Mexico. We determined the prevalence of bacterial enteropathogens in 831 children with acute diarrhoea over a four-year period in Yucatan, Mexico. Six DEC supplementary virulence genes (SVG), mainly associated with enteroaggregative *E*. *coli* (EAEC), were sought in 3100 *E*. *coli* isolates. DEC was the most common bacterial enteropathogen (28%), surpassing *Salmonella* (12%) and *Shigella* (9%). Predominant DEC groups were diffusely adherent *E*. *coli* (DAEC) (35%), EAEC (24%), and enteropathogenic *E*. *coli* (EPEC) (19%). Among children with DEC infections, 14% had severe illness mainly caused by EPEC (26%) and DAEC (18%); 30% had moderate diarrhoea mainly caused by DAEC (36%), mixed DEC infections (33%) and EAEC (32%). DAEC was most prevalent during spring, while ETEC, EAEC and EPEC predominated in summer. EAEC was more frequent in children 6–24 months old than in those younger than 6 months of age (P = 0.008, OR = 4.2, 95% CI, 1.3–13.9). The presence of SVG dispersin, (*aatA*), dispersin-translocator (*aatA*), enteroaggregative heat-stable toxin 1 (*astA*), plasmid encoded toxin (*pet*), cytolethal distending toxin (*cdt*) was higher in DEC than non-DEC strains, (36% vs 26%, P <0.0001, OR = 1.5, 95% CI, 1.3–1.8). 98% of EAEC-infected children harboured strains with SVG; 85% carried the *aap-aatA* gene combination, and 33% of these also carried *astA*. 28% of both EPEC and ETEC, and 6% of DAEC patients had strains with SVG. 54% of EPEC patients carried *pet*-positive strains alone or in combination with *astA*; only this DEC group harboured *cdt*-positive isolates. All ETEC patients carried *astA*- or *astA-aap*-positive strains. *astA* and *aap* were the most common SVG in DAEC (3% and 2%) and non-DEC strains (21% and 13%). DEC carrying SVG are an important cause of moderate to severe bacterial diarrhoea in Mexican children.

## Author Summary

Diarrhoea is an important cause of illness and death among young children in low- and middle-income countries. Nonetheless, very few epidemiological studies of diarrhoea have been conducted in Mexico during the last two decades. In recent years, a group of bacteria known as diarrheagenic *Escherichia coli* (DEC) have been recognized as a major cause of diarrheal illness worldwide. This group cannot be identified by the conventional biochemical methods used for other diarrhoeal pathogens such as *Salmonella* or *Shigella*, which limits its identification. We conducted a study in Yucatan, Mexico, to identify the bacterial causes of acute diarrhoea in children less than five years of age who required hospitalization. All DEC strains were further characterized for the presence of six *E*. *coli* supplementary virulence genes (SVG). Of the 831 children with acute diarrhoea, a bacterial pathogen was found in 56%. DEC was the most prevalent (28%) pathogen, surpassing *Salmonella* and *Shigella*. Among children with DEC diarrhoea, 44% were moderately or severely ill. Thirty-six percent of the DEC strains had one or more SVG, which were more common in older children. Our results strongly suggest that DEC carrying SVG are an important cause of moderate to severe bacterial diarrhoeal disease in Mexico.

## Introduction

Diarrhoeal illness is a major cause of mortality and morbidity among children less than 5 years of age accounting for 711,800 deaths and 1.731 billion cases, with an incidence of 2.7 diarrhoeal episodes per child per year in 2011 [1]. In Mexico, diarrhoeal illness continues to be a national health problem [2,3]. It is the second cause of morbidity and the fifth cause of mortality among children under five years of age. In 2010, more than 5.5 million diarrhoeal cases were reported for a national rate of 5,264.24 per 100,000 inhabitants; most of these cases occur in young children [2,3]. The burden is particularly high in the state of Yucatan, which occupies third place in diarrhoea-associated mortality nationwide [3].

The prevalence of different enteric pathogens varies among countries and geographical areas [4–9]. The public health importance of the diarrheagenic *Escherichia coli* groups (DEC) has acquired greater relevance in recent years [10–14]. DEC infections have been associated with acute and persistent diarrhoea (>14 days), as well as with growth delays and stunting, which in turn can lead to long-term cognitive impairment [15–17]. Moreover, several studies suggest that DEC is responsible for up to 30%-40% of acute diarrhoeal episodes in children [18].

DEC have been traditionally classified in six groups based on clinical, epidemiological and virulence traits: enterotoxigenic *E*. *coli* (ETEC), typical and atypical enteropathogenic *E*. *coli* (tEPEC, aEPEC), enteroinvasive *E*. *coli* (EIEC), enteroaggregative *E*. *coli* (EAEC), diffusely adherent *E*. *coli* (DAEC) and Shiga-toxin—producing *E*. *coli* (STEC) [10,19]. ETEC produces thermo labile (Lt) and thermo stable (St) toxins and STEC produces shiga-toxins 1 (Stx1) and 2 (Stx2). EIEC invades eukaryotic cells, and EPEC and some STEC strains induce cell transmembrane signalling through intimin and its translocated intimin receptor, leading to the formation of attaching and effacing (A/E) lesions. In contrast, DAEC and EAEC strains continue to be identified by their characteristic adherence patterns on HEp-2 cells and not by production of known toxins or molecular markers associated with virulence [19]. Similarly, aEPEC strains are defined solely by the presence of intimin and the absence of both bundle forming pilus (bfp) and shiga toxins [18]. Although the latter three DEC groups have been associated with acute as well as protracted and persistent diarrhoea, [10,11,20–22], there is not yet a consensus on the specific virulence markers for these strains; since the adherence properties or presence of specific genes alone do not always distinguish between pathogenic and non-pathogenic isolates [14,23,24]. In light of the high prevalence of DEC strains among ill children and adults worldwide, there is a clear need for identifying the key virulence genes associated with illness.

Aside from the virulence genes that define each DEC, several others have been reported, particularly in EAEC. Dispersin, dispersin Aat translocator system, plasmid encoded toxin (Pet) and enteroaggregative heat-stable toxin 1 (EAST1), for example, were first described in EAEC strains [11,23–25]. Dispersin, an anti-aggregation secreted protein, which acts to disperse the bacteria through the mucus layer produced by the host in response to EAEC infection, is translocated by the EAEC anti-aggregation transporter protein (Aat) [26,27]. Pet induces contraction of the cytoskeleton [28] and EAST 1 stimulates the guanylate cyclase receptor in a similar fashion to both ETEC St toxin and guanylin [25]. Two toxins that cause apoptosis have been identified in other DEC groups. Cytolethal distending toxin (CDT), that induces an irreversible cell cycle arrest [29], and Subtilase cytotoxin (*SubAB*), which provokes severe and unresolved endoplasmic reticulum stress, have been described, respectively, in EPEC and a subset of STEC strains [30].

Despite the fact that diarrheal illness continues to be a major cause of death and morbidity in Mexican children, few molecular epidemiology studies on DEC have been conducted [21,31,32]. As a consequence, the true impact of DEC as an agent of acute diarrhoea in Mexico is unknown. We recently showed that EAEC and aEPEC were the most frequent DEC groups identified in children with diarrhoea from Yucatan, Mexico [33]. In the present study, we determined the prevalence of DEC among bacterial enteropathogens (*Shigella*, *Salmonella*, *Vibrio* and *Campylobacter*) recovered from children with acute diarrhoea in Yucatan, as well as the presence of supplementary virulence genes in DEC strains. We also determined the clinical severity and age distribution in children with DEC-associated diarrhoea. As well as the distribution of DEC groups diarrhoea episodes by seasonality, rainfall and temperature

## Materials and Methods

### Ethics statement

This study was approved by both the Hospital O’Horan Ethics Committee and the CINVESTAV Committee of Bioethics for Human Research. Legal guardians of the children involved in this study were required to sign an informed consent form. All children received medical treatment for their diarrhoea according to the hospital protocols.

### Study site and patients

The study was conducted at the Hospital General O’Horan, a tertiary-care public hospital located in Yucatan, southeast Mexico. Yucatan has a warm and humid climate due to its proximity to the Gulf of Mexico. The average annual high temperature is 33°C (91°F), ranging from 28°C (82°F) in January to 36°C (97°F) in May, but temperatures often rise above 38°C (100°F) at this time. Low temperatures range between 18°C (64°F) in January to 23°C (73°F) in May and June. The rainy season runs from June through October. The average yearly rainfall is 115 centimetres (45 inches), being heaviest during the summer months.

After written informed consent was obtained from the children’s guardians, stool samples were collected from children with acute community-acquired diarrhoea requiring hospitalization in the Paediatric Emergency Room. Diarrhoea was defined as three or more liquid or semiliquid stools passed in a 24 h period. Severity of disease was based on the World Health Organization classification of dehydration [34]. Children with no dehydration were classified as mild diarrhoea and those with mild to moderate dehydration were classified as moderate diarrhoea. Dysenteric syndrome was also classified as moderate based on the experience at our hospital that no episodes are caused by *Shigella dysenteriae* Type 1 [35]. Children who required intravenous therapy for correction of dehydration and/or electrolyte disturbance were classified as severe.

### Stool samples and bacteriological analysis

Stool samples were collected in sterile containers containing Cary Blair media and analysed for the presence of *Salmonella* spp, *Shigella* spp, *Escherichia coli*, *Campylobacter* spp, and *Vibrio* spp. by standard procedures [36]. Briefly, samples were inoculated onto XLD, Hektoen Enteric, Brilliant Green, Cefex, TCBS and MacConkey agars as well as Tetrathionate, Rappaport and alkaline peptone broths, and incubated at 37°C for 18–24 h. Broths were subcultured to XLD, Brilliant Green and TCBS agar plates and incubated for another 18–24 h. Identification of *Salmonella*, *Shigella* and *Vibrio* isolates were performed with conventional biochemical tests and confirmed with API 20E strips (BioMerieux, Marcy l’Etoile, France). *Campylobacter* isolates were confirmed morphologically with phase-contrast microscopy and the oxidase and catalase tests. Five *E*. *coli*-like colonies were selected from each MacConkey agar plate, characterized by conventional biochemical tests and subjected to PCR testing.

### PCR assays for the identification of diarrheagenic *E*. *coli*

For the purposes of this study, DEC was defined as an *E*. *coli* strain that contained the set of defining genes for a specific group as described in the scientific literature, hereby referred to as “DEC-defining gene” [19,21,37,38]. A non-DEC strain was an *E*. *coli* strain that did not contain the set of defining genes but could harbour a different virulence gene, hereby referred to as “supplementary virulence gene”. All *E*. *coli* isolates were subjected to several PCR assays to identify DEC-defining genes and supplementary virulence genes (SVG). For all PCR assays, bacterial lysates were prepared by resuspending a single colony in 1 ml of deionized water (MilliQ) in a polypropylene tube, followed by boiling for 1 min. Two previously described multiplex PCR assays [37,39] were used for the identification of DEC-defining genes (Table 1). PCR 2 also identifies two of the SVG, *aap* and *aatA* genes encoding for dispersin and its Aat translocator, previously known as AA probe, respectively [38]. STEC strains were further characterized for the expression of the O157 lipopolysaccharide antigen and enterohemolysin gene (*hlyA*) using a latex particle agglutination kit (Oxoid Limited, Basingstoke, UK), and PCR, respectively.

**Table 1:** Phenotypic and genotypic characteristics used to identify of diarrheagenic *Escherichia coli* (DEC) groups in this study.

DEC group	Defining characteristic(s)	Target genes	Multiplex PCR assay
**tEPEC**	Presence of both intimin (as a marker of the pathogenic island LEE) and the BFP contained in the EAF plasmid	*eaeA*, *bfpA*	1
**aEPEC**	Presence of intimin (as a marker of LEE); absence of the EAF plasmid and Shiga toxins 1 and 2	*eaeA*	1
**ETEC**	Presence of thermo labile (LT) or/and thermo stable (ST) toxins	*lt*, *st*	1
**EIEC**	Presence of the invasion-associated locus (IAL) of the invasion plasmid	*ial*	1
**STEC**	Presence of Shiga toxin 1 (STX1) and/or 2 (STX2); in addition, some strains also have intimin (as a marker of LEE)	*stx1*, *stx2*, *eaeA*	1
**EAEC**	Presence of AggR master regulon most genes associated with the aggregative adherence (AA) and EAEC virulence are controlled by this regulon.	*aggR*	2
**DAEC**	Presence of surface afimbrial adhesins as AfaE-I and AfaE-III, that are encoded on the Afa/dr/daa operon	*afaC*	2

ETEC: enterotoxigenic *E*. *coli*,

tEPEC: typical enteropathogenic *E*. *coli*,

aEPEC: atypical enteropathogenic *E*. *coli*,

EAEC: enteroaggregative *E*. *coli*,

DAEC: diffusely adherent *E*. *coli*.

LEE: locus of enterocyte effacement,

BFP: bundle-forming pilus;

EAF: EPEC adherence factor plasmid, *eaeA* and *afaC*: genes encoding for intimin and Afa fimbria usher, respectively.

### Development of a PCR assay for the identification of four *E*. *coli* toxin genes

**Fig 1:**
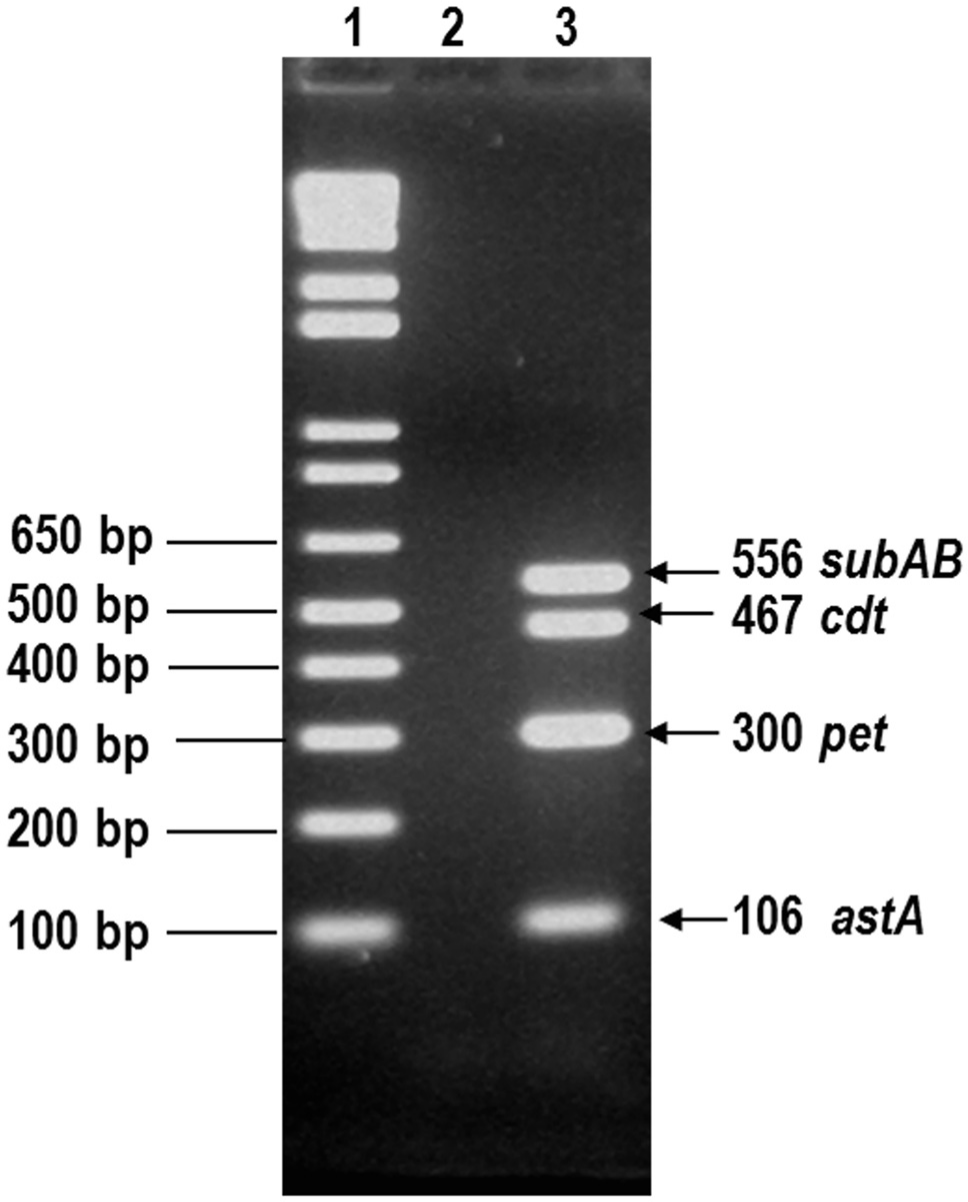
PCR products. Lane1, 1-kb molecular weight marker in base pairs; lane 2, negative control and lane 3, PCR products obtained with DNA of reference strains mix EAEC 042 (*astA*, *pet*) and EPEC O86:H34 (*cdt*), and DNA from the strain 98NK2 (*SubAB*)

We developed a third multiplex PCR to identify four other *E*. *coli* SVG using previously described primers for *astA* [40], *cdt* [41], *pet* [42] and *SubAB* [43] that encode EAST1, CDT, Pet and Subtilase toxins, respectively. For standardization purposes, we used DNA from reference strains EAEC 042 (*astA*, *pet*) and EPEC O86:H34 (*cdt*). We also used DNA from strain 98NK2 (*SubAB*), provided by Dr. Pablo Okhuysen (University of Texas, Houston). Best results were obtained using a mixture of primers in the following concentrations: 16 pMol for *astA* primers, 6 pMol for *pet*, 10 pMol for *SubAB* and 5 pMol for both *cdt1* and *cdt2* set of primers. Briefly, each PCR tube contained 21 μl of reaction mix of the following concentrations: 2 mM Tris-HCl (pH 8.0), 10 μM EDTA, 100 μM DTT, 50% (vol/vol) glycerol, 1.6 mM MgCl_2,_ 0.75U/25 μl Ampli Taq polymerase (Invitrogen) and 200 μM each dATP, dCTP, dGTP and dTTP (Invitrogen); 2 μl of the primer mix and 2 μl of the bacterial lysate. The PCR tubes were then subjected to the following cycling conditions: 95°C (1 min, 1 cycle); 95°C, 62°C (1 min at each temperature) and 72°C, 1 min 20 s all for 35 cycles; and a final extension step (10 min, 72°C) in a thermal cycler (Bio-Rad). 5μl of PCR products were visualized after electrophoresis in a 3% agarose gel in Trisacetate-EDTA buffer and ethidium bromide staining. Only the presence of the correct sized gene product(s) was interpreted as a positive test. Fig. 1 illustrates the amplicon sizes of PCR products for all 4 loci using a DNA control mix that contained 1.5 μl of bacterial lysate from 042 and O86:H34 strains, plus 0.5 μl of subtilase DNA in a final concentration of 0.212 μg/μl.

### Statistical analysis

Groups were compared by a two-tailed chi-square test. When analysis was constrained by low expected values, Fisher’s exact test was employed. Odds ratio (OR) and 95% confidence intervals (CIs) for the likelihood of co-detection were calculated using GraphPad PRISM^®^version 5.0b software.

## Results

### Prevalence of bacterial enteropathogens and DEC groups

**Fig 2:**
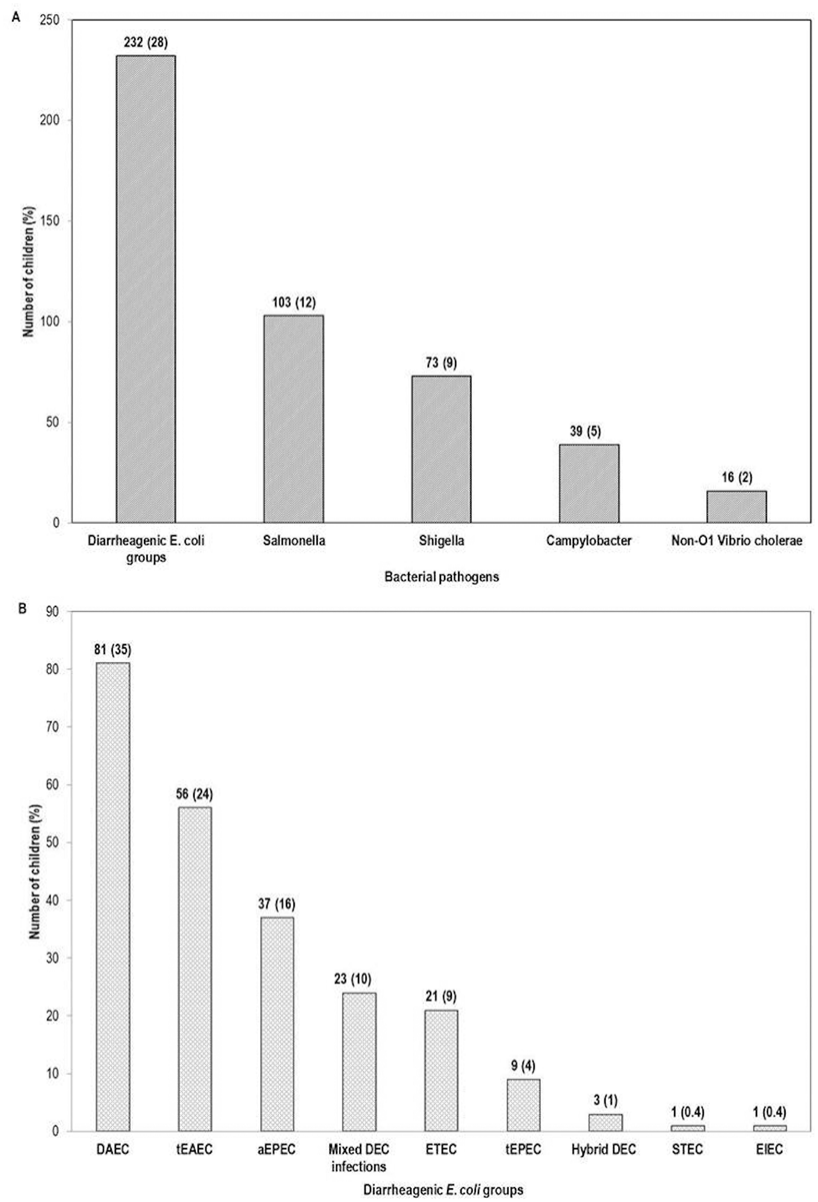
(A) Prevalence of bacterial enteropathogens among 831 children with acute diarrhoea in Yucatan, Mexico. (B) Distribution of diarrheagenic *Escherichia coli* (DEC) groups, mixed DEC and hybrid DEC infections in 232 DEC positive-children. ETEC: enterotoxigenic *E*. coli, tEPEC: typical enteropathogenic *E*. *coli*, aEPEC: atypical enteropathogenic *E*. *coli*, tEAEC: typical enteroaggregative *E*. *coli*, DAEC: diffusely adherent *E*. *coli*, mixed DEC: more than one DEC group strain in the same patient and hybrid DEC: a patient with an *E*. *coli* strain with two or more DEC defining genes.

A total of 831 children less than five years of age were recruited from January 2007 to January 2009 and from January 2010 to December 2011. One or more bacterial enteropathogens were identified in 463 (56%) children; DEC was the most prevalent bacterial group (28%), followed by *Salmonella* spp (12%), while non-O1 *Vibrio cholerae* (2%) was the least prevalent (Fig. 2A). Of the 232 children with DEC, 61 were co-infected with other bacterial pathogens (*Salmonella*, 27; *Shigella*, 24; *Campylobacter*, 6; *Vibrio*, 4).

The distribution of DEC groups isolated from 232 children is shown in Fig. 2B; a large proportion of these harboured DAEC (35%) and EAEC groups (24%) whereas EIEC and STEC groups were rare. None of the STEC strains were O157. The majority of children had single DEC infections (infections in which only one DEC group was isolated from the patient’s stool). Ten percent of patients had mixed DEC infections (more than one DEC group in the same patient). Of the 23 children with mixed infections, six harboured DAEC+EAEC strains, five DAEC+ETEC, five EAEC+ETEC and three DAEC+aEPEC. In addition, EAEC+aEPEC, ETEC+aEPEC and ETEC+tEPEC strains were identified in one patient each, and one child was infected with three different DEC strains (DAEC+EAEC+ETEC). Another three children (1%) had hybrid infections caused by *E*. *coli* strains carrying defining genes for more than one DEC; two patients carried a DAEC-EAEC strain and one child carried an aEPEC-ETEC strain.

In most patients with single DEC infections, at least three of the five *E*. *coli* strains collected per patient were positive for DEC-defining genes. This was observed in 67%, 64%, 62.5%, 62% and 59% of patients positive for tEPEC, DAEC, EAEC, aEPEC and ETEC, respectively.

### Distribution of DEC groups by age

Of the 831 children, 17% were younger than 6 months, 31% were between 6–12 months, 32% were between 13–24 months and 20% were older than 24 months. DEC diarrhoeal episodes occurred with similar frequency in all age groups: 23%, 30%, 27% and 31%, respectively (Table 2). There were statistically significant associations between age and infection with a specific DEC group as follows: 1) EAEC diarrhoea was more frequently observed among children in the 6–24 months age group than children less than six months of age (8.3% vs. 2.1%, *P* = 0.008, OR = 4.2, 95% CI, 1.3–13.9) and 2) children in the 13–24 months age group had less mixed DEC infections than children in the other age groups (0.7% vs. 3.7%, *P* = 0.012, OR = 0.19, 95% CI, 0.04–0.8) (Table 2). A trend was observed for children younger than 6 months old to be more frequently infected with tEPEC strains (2.8% vs. 0.72%, *P* = 0.052, OR = 4.2 95% CI, 1.3–14).

**Table 2:** Distribution of DEC groups by age in hospitalized children with diarrhoea in Yucatan, Mexico.

		Children’s age in months			
	Patients (%)	<6	6–12	13–24	>24
	831	143 (17)	255 (31)	270 (32)	163(20)
**Total DEC (%)**	232 (28)	33 (23)	77 (30)	72 (27)	50 (31)
**DAEC infections (%)**	81 (35)	10 (7)	21 (8.2)	28 (10.4)	22 (13.5)
**EAEC infections (%)**	56 (24)	3 (2.1)	24 (9.4)	20 (7.4)	9 (5.5)
**aEPEC infections (%)**	37 (16)	7 (4.9)	13 (5.1)	12 (4.4)	5 (3.1)
**Mixed DEC infections (%)**	23 (10)	4 (2.8)	9 (3.5)	2 (0.7)	8 (4.9)
**ETEC infections (%)**	21 (9)	4 (2.8)	7 (2.7)	7 (2.6)	3 (1.8)
**tEPEC (%)**	9 (4)	4 (2.8)	2 (0.8)	2 (0.7)	1 (0.6)
**Hybrid DEC infections (%)**	3 (1)	1 (0.7)	0	0	2 (1.2)
**STEC infections (%)**	1 (0.4)	0	1(0.4)	0	0
**EIEC infections (%)**	1 (0.4)	0	0	1 (0.4)	0

ETEC: enterotoxigenic *E*. *coli*,

tEPEC: typical enteropathogenic *E*. *coli*,

aEPEC: atypical enteropathogenic *E*. *coli*,

EAEC: enteroaggregative *E*. *coli*,

DAEC: diffusely adherent *E*. *coli*.

### Distribution of DEC groups by seasonality, rainfall and temperature

**Fig 3:**
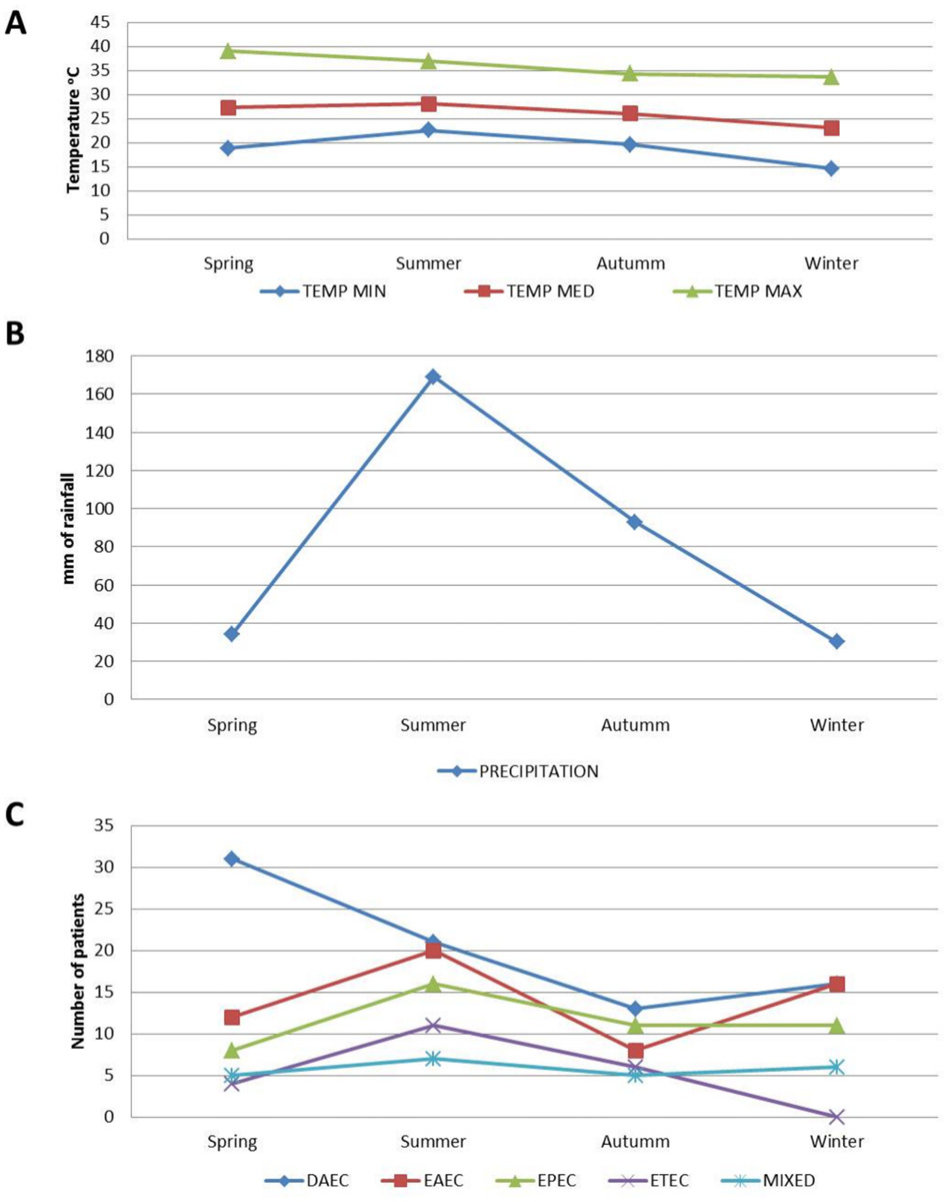
Distribution of DEC groups, over a four-year period, by season according to yearly average precipitation and minimum, maximum, and average atmospheric temperature in Yucatan Mexico. DAEC: diffusely adherent *E*. *coli*, EPEC: enteropathogenic *E*. *coli*, EAEC: enteroaggregative *E*. *coli*, ETEC: enterotoxigenic *E*. *coli* and MIXED DEC.

The distribution of DEC groups by seasonality, rainfall and temperature for the four-year period (average) is shown on Fig. 3. During all four years DAEC cases were more prevalent in spring (31/81, 38%), the season with the highest temperatures and the lowest rainfall (average precipitation = 34.4 mm). In contrast, ETEC (11/21, 52%) EAEC (20/56, 36%) and EPEC (16/46, 35%) were more prevalent during the summer, which is the rainy season (average precipitation = 169 mm) in Yucatan.

### Severity of DEC diarrhoeal episodes

Complete clinical data were available for 126 children with single or mixed DEC infections (no other bacterial pathogen). Eighteen children (14%) had severe diarrhoea requiring intravenous rehydration and 14 (11%) had bloody diarrhoea with fever (DAEC, 7; EAEC, 5 and EPEC, 2). Severe diarrhoeal episodes were more frequently observed in children with EPEC (26%) and DAEC (18%). Most children (71%) were hospitalized for less than 24 hours, but 7 (6%) were admitted for more than 72 hours (Table 3).

**Table 3:** Severity of disease and length of hospitalization in children by DEC group.

	Length of hospitalization n (%)^1^			Severity of diarrheal episode n (%)^1^		
**DEC group**	< 24 h	24–72 h	>72 h	Mild	Moderate	Severe
**DAEC (n = 50)**	32 (64)	15 (30)	3 (6)	23 (46)	18 (36)	9 (18)
**EAEC (n = 31)**	26 (84)	5 (16)	0 (0)	20 (65)	10 (32)	1 (3)
**EPEC (n = 23)**	14 (61)	6 (26)	3 (13)	13 (57)	4 (17)	6 (26)
**ETEC (n = 10)**	8 (80)	2 (20)	0 (0)	7 (70)	2 (20)	1 (10)
**Mixed DEC infection (n = 12)**	9 (75)	2 (17)	1 (8)	7 (58)	4 (33)	1 (8)
**Total (n = 126)**	89 (71)	30 (24)	7 (6)	70 (56)	38 (30%)	18 (14%)

^1^Refers to percentage of patients for each DEC group and total DEC infections

### Distribution of supplementary virulence genes among DEC and non-DEC strains

A total of 3100 *E*. *coli* strains were isolated from 621 (75%) of the children’s’ stool samples; 840 DEC strains were isolated from 232 children of which 298 (36%) had one or more supplementary virulence genes (SVG). Two thousand sixty non-DEC *E*. *coli* strains were isolated from the remaining 389 children; 589 (26%) of these, isolated from 147 children, carried SVG. DEC isolates had significantly more SVG than non-DEC strains, 36% vs. 26%, (*P* <0.0001, OR = 1.5, 95% CI, 1.3–1.8).

The distribution of DEC and non-DEC strains carrying SVG is shown on table 4. Neither the five (0.6%) EIEC or three (0.35%) STEC strains harboured SVG and only one of the 9 hybrid isolates carried two of these genes. As shown in Table 4, of the remaining 297 DEC strains with SVG, the EAST 1 toxin gene (*ast*A) was identified in all 4 DEC groups, and was more frequently found among EAEC, ETEC and tEPEC isolates than in aEPEC and DAEC strains (*P* <0.0001, OR = 9, 95% CI, 6–15). In contrast, the *aap* gene encoding for dispersin was significantly more common in EAEC than ETEC or DAEC (*P* <0.0001, OR = 214, 95% CI, 63–728, OR = 449, 95% CI, 181–1113, respectively), and was not found in any EPEC strains. Furthermore, the *aatA* gene encoding for the dispersin Aat translocator system was only identified in EAEC isolates. Among the 208 EAEC strains carrying SVG, the *aap*-*aatA* gene combination was the most frequently identified (85.5%). Strains harbouring the gene for Pet toxin (*pet*) were only identified in EAEC and EPEC groups, more frequently among the latter group (*P* = 0.0183, OR = 2.5, 95% CI, 1–6). The gene encoding for CDT toxin (*cdt*) was exclusively found in EPEC isolates, mainly in tEPEC (*P* < 0.0001, OR = 20.3, 95% CI, 4.1–99.1). Of note, only 14 (4%) DAEC strains harboured SVG.

**Table 4:** Distribution of supplementary virulence genes (SVG) among Diarrheagenic *E*. *coli* (DEC) and non-DEC strains isolated from children with moderate to severe acute diarrhoea from Yucatan, Mexico.

*E*. *coli* strains, N	Strains with SVG n (%)	Supplementary virulence genes n (%)				
**DEC group, 824**	297 (36)	*aap*	*aatA*	*astA*	*Pet*	*cdt*
**EAEC, 223**	208 (93)	198 (89)	183 (82)	77 (34.5)	10 (4.5)	0
**tEPEC, 38**	20 (53)	0 (0)	0 (0)	11 (29)	1 (3)	9 (38)
**aEPEC, 133**	29 (22)	0 (0)	0 (0)	13 (10)	18 (13.5)	2 (1.5)
**ETEC, 84**	26 (31)	3 (3.5)	0 (0)	25 (30)	0 (0)	0 (0)
**DAEC 346**	14 (4)	6 (2)	0 (0)	11 (3)	0 (0)	0 (0)
**Non-DEC, 2260**	589 (26)	302 (13)	30 (1.3)	477 (21)	30 (1.3)	3 (0.6)

ETEC: enterotoxigenic *E*. *coli*,

tEPEC: typical enteropathogenic *E*. *coli*,

aEPEC: atypical enteropathogenic *E*. *coli*,

EAEC: enteroaggregative *E*. *coli*,

DAEC: diffusely adherent *E*. *coli*.

*aap* and *aatA*: genes encoding for dispersin and its translocator,

*astA*, *pet* and *cdt*: genes encoding for enteroaggregative heat-stable toxin 1, plasmid encoded toxin and cytolethal distending toxin, respectively.

*astA* was the most prevalent SVG among non-DEC strains (21%). This prevalence rate was lower than those found in EAEC, ETEC and tEPEC isolates but higher than in aEPEC and DAEC (*P* < 0.0001, OR = 5, 95% CI, 3.3–7.7). Both *aap* and *aatA* genes were significantly more frequent in EAEC isolates than in non-DEC strains, (aap: 89% vs.13%, *P* < 0.0001, OR = 51.3, 95% CI, 33.3–79.2; aatA: 82% vs.1.3%, *P* < 0.0001, OR = 340, 95% CI, 207–559). In contrast, of the 589 non-DEC strains carrying SVG, only three (0.5%) carried the *aap*-*aatA* gene combination. Positive-*pet* strains were more often found in EPEC than non-DEC, 11% vs. 1.3%, respectively, (*P* < 0.0001, OR = 9.3, 95% CI, 5.1–16.9). Similarly, *cdt*-positive strains were found among 6.4% EPEC isolates vs 0.6% of non-DEC strains (*P* < 0.0001, OR = 11.8, 95% CI, 5.2–26.9).

### Children with single DEC infections and SVG-carrying strains

As shown in Table 5, of the 56 children infected with EAEC, 54 (96%) harboured strains with SVG; the only two children whose isolates did not carry any SVG were younger than 12 months. Most children (85%) had EAEC strains that simultaneously carried the genes for dispersin and its translocator (*aap*-*aatA*). These strains were distributed among all age groups, whereas *pet*-positive EAEC strains were only found in the 13–24 months group. Aside from EAEC strains, no other DEC strains carried the *aap*-*aatA* gene combination. The percentage of children harbouring tEPEC (33%) and aEPEC (27%) strains with SVG was similar and there was a clear trend for the presence of SVG with increasing age (Table 5). tEPEC strains harbouring SVG were significantly more common in children older than 24 months compared with those younger than 24 months (66% vs. 29%) (*P* = 0.023, OR = 8.8, 95% CI, 1.4–56.2). Furthermore, patients harbouring *cdt*-positive EPEC strains were exclusively found in children older than 24 months. No associations with age were observed for SVG in ETEC and DAEC.

**Table 5:** DEC strain gene profiles and their distribution by age group.

	Gene profile	Age in months			
**DEC Patients**		< 6	6–12	13–24	> 24
		n = 33	n = 77	n = 72	n = 50
**DEC Patients with SVG (%)**		7(21)	27(35)	28 (39)	16(32)
**EAEC, 54/56 (96)**		2 (66)	23(92)	20(100)	9(100)
	a*ggR-aap*	0	1	0	0
	a*ggR-astA*	0	0	1	0
	a*ggR-aap*-*aatA*	1	12	5	6
	a*ggR-aap*-*astA*	0	0	2	2
	a*ggR-aap*-*pet*	0	0	1	0
	a*ggR-aatA*-*astA*	0	1	0	0
	a*ggR-aap*-*aatA*-*astA*	1	9	7	1
	a*ggR-aap*-*aatA*-*astA*-p*et*	0	0	4	0
**tEPEC, 3/9 (33)**		2 (50)	0 (0)	0 (0)	1 (100)
	*bfp*-*eaeA*-*astA*	1	0	0	0
	*bfp*-*eaeA-astA-pet*	1	0	0	0
	*bfp*-*eaeA-cdt*	0	0	0	1
**aEPEC, 10/37 (27)**		1 (14)	2 (15)	4 (33)	3 (60)
	*eaeA-astA*	0	0	2	1
	*eaeA-astA-pet*	1	1	0	0
	*eaeA-pet*	0	1	2	1
	*eaeA-cdt*	0	0	0	1
**ETEC, 6/21 (28)**		1 (25)	1 (12)	2 (33)	2 (66)
	*lt-astA*	1	0	0	1
	*lt-st-astA*	0	1	1	0
	*st-aap-astA*	0	0	1	1
**DAEC, 5/81 (6)**		1 (10)	1 (5)	2 (7)	1 (4)
	*afa-aap*	0	0	1	1
	*afa-astA*	1	1	1	0

ETEC: enterotoxigenic *E*. *coli*,

tEPEC: typical enteropathogenic *E*. *coli*,

aEPEC: atypical enteropathogenic *E*. *coli*,

EAEC: typical enteroaggregative *E*. *coli*,

DAEC: diffusely adherent *E*. *coli*.

*aap* and *aatA*: genes encoding for dispersin and its translocator,

*astA*, *pet* and *cdt*: genes encoding for enteroaggregative heat-stable toxin 1, plasmid encoded toxin and cytolethal distending toxin, respectively.

## Discussion

Despite the plethora of scientific reports on diarrheal disease in children under five years of age, this study is one of the few to include the identification of routine bacterial pathogens and all six DEC groups by molecular methods in a hospital setting over a four year period. In our active surveillance study that included children with acute diarrhoea in Yucatan, bacterial pathogens were found in a higher proportion (56%) of patients than in other tropical regions [9,44]. Recent studies on diarrheal disease conducted at Paediatric hospitals in Brazil [9] and Burkina Faso [44], for example, reported a prevalence of bacterial pathogens of 42% and 40%, respectively. This contrasts with a 26% prevalence reported at a Paediatric Emergency Department in Cincinnati [5]. A common denominator in all these studies was that DEC, as a group, was the most frequently identified bacterial pathogen in children with diarrhoea. Possible explanations for these geographic differences include variations in socioeconomic conditions, laboratory infrastructure and climate. Our study found strong seasonal patterns for almost all DEC-groups. DAEC was highly prevalent during the hot, dry months, while ETEC, EAEC and EPEC were most frequent during the rainy season, which s with other studies conducted in Mexico [21,45].

Unexpectedly, DAEC was isolated from 10% of our study subjects with diarrhoea, followed by EAEC (7%) and aEPEC (4.5%). Spano et al., found that DAEC was the top DEC group (18.3%) at a Paediatric Emergency Room in Brazil [9], followed by aEPEC (5.5%) and EAEC (4.6%). Likewise, in a cohort study conducted in children from a periurban area of Lima, Peru, Ochoa et al., [7] identified EAEC as the most frequent DEC (15.1%) followed by EPEC (7.6%) and DAEC (4.6%), respectively. The role of ETEC as an aetiologic agent of diarrhoeal disease in Latin American children appears to be decreasing [7,21, 46]. Only 2.5% of our study subjects had ETEC diarrhoea compared to 3.2% in the Brazilian and Peruvian studies. With the exception of Argentina [47] and Chile [48], STEC continues to be uncommon in most Latin American countries. Only one child (0.1%) in our study had this DEC group compared to 0% and 0.5% in Brazil and Peru, respectively [7,9].

Our results, as those of other investigators, clearly show that DEC groups are major aetiologic agents of diarrheal disease in children [10–14]. Almost half of the children with DEC-associated diarrhoea in this study were moderately to severely ill. Notably, 11% presented bloody diarrhoea with fever, most frequently in DAEC-infected children. DAEC strains producing bloody diarrhoea has been reported by others [7]. We recently reported the case of a young child with prolonged bloody diarrhoea associated with DAEC in which we hypothesized that this clinical presentation could have been caused by disassembly of tight junction-associated proteins and cytokine induction [39].

Studies conducted in endemic areas have reported isolation of DEC strains, particularly EAEC, DAEC and aEPEC, in similar proportions from both children with diarrhoea and healthy controls [11, 14, 21,49]. These studies, however, only report presence or absence of DEC. A recent study, that measured DNA by quantitative real-time PCR from EPEC-positive stools from children with and without diarrhoea [50], showed that the EPEC load was significantly higher in sick children, thus demonstrating that high bacterial load correlates with illness. Although bacterial load was not determined in the present study, we observed that most DEC patients had three or more *E*. *coli* strains carrying DEC-defining genes out of the 5 strains that were selected per patient. A possible correlation between the proportion of DEC-positive strains per patient and DEC bacterial load in faeces remains to be established. Greater severity of illness was observed in DAEC and EPEC-associated diarrhoea. In these DEC groups, severity might be related to the higher frequency of bloody diarrhoea, and severe dehydration, respectively.

Our study revealed interesting associations between specific DEC groups and age. EAEC diarrhoea, for example, was more common in children 6–24 months of age than those in younger or older age groups. In the Peruvian cohort study [7], EAEC diarrhoea was also higher in children older than 6 months. Children younger than six months might be protected from DEC infection by breastfeeding and be less exposed to bacterial pathogens since most have limited introduction to solid foods and have not yet started to crawl. EAEC was also more frequently isolated from children less than 2 years in India [12]. The lower prevalence in older children is likely due to age-related immunity, which we have observed for other pathogens such as *Salmonella* and *Campylobacter* in our population [51,52].

Since most of the SVG sought in this study were first identified in EAEC and EPEC as expected, they were extremely frequent in our EAEC (93%) and tEPEC (53%) strains. The SVG were also present in about a quarter of our ETEC (31%), aEPEC (22%) and non-DEC (26%) strains. The EAST1 encoding gene was widely distributed in all four DEC groups. Since EAST1 shares chemical and functional similarities with the ETEC STa [23], it has been suggested that *astA*-carrying strains could have increased ETEC virulence [53]. The *astA* gene has been identified in ETEC strains possessing major adhesins that are pathogenic for humans [53]. Furthermore, most recent cases of ETEC diarrhoea in Latin America, including Mexico, have been associated with *lt*-positive strains [7, 9,21,31] whereas *st*-positive isolates were predominant in the past [46,54]. An increased virulence conferred by EAST1 could explain this epidemiological shift. *astA*-positive isolates of EAEC [55,56] and aEPEC [57,58] have also been associated with diarrhoea.

Eighty percent of our EAEC strains simultaneously harboured *aap-aat*A, the genes encoding for dispersin and its AatA translocator system. Our results concur with previous findings for EAEC isolates collected from other regions of Mexico [31] and the world [45]. There appears to be a high conservation of the *aggR*-*aap-aat*A locus in the pAA large virulence plasmid, as has been shown for the prototype 042 strain [59]. Among DEC isolates, the *aatA* gene was solely found in EAEC strains, (82%), and in 1.3% of non-DEC strains. The *aap*-*aatA* combination, however, was only found in 0.17% of non-DEC isolates. *aap*-positive strains were significantly more frequent among EAEC isolates than in other DEC or non-DEC strains (*P* <0.0001). It remains to be shown if dispersin is actually secreted by non-EAEC DEC strains. If so, we may assume that it would not be through the Aat translocator system, since none of these strains carried the *aatA* gene.

*cdt*-positive strains were only identified in our EPEC patients as has been described by others [60,61]. Although Pet toxin was first described in EAEC strains, *pet* was the main SVG identified in aEPEC patients, a feature that may increase their virulence. Of interest, children harbouring tEPEC and aEPEC strains carrying SVG were more frequently found among patients older than 24 months of age. Since children in highly endemic settings acquire protective immunity to diarrhoeal illness around 24 months, it is likely that pathogens would need an increasing number of virulence genes to cause disease in older hosts.

A significant proportion of our non-DEC strains had SVG (26%); of these, 86.5% carried a gene encoding for a toxin, most frequently *astA*. Forty-three percent carried more than one supplementary gene and 13% of non-DEC strains carried the *aap* gene. Non-DEC strains have also been associated with diarrhoea in Japanese children [61,62] strongly suggesting that these strains have the potential to cause illness.

The presence of SVG in both DEC and non-DEC strains are indicative of continuous horizontal gene transfer between *E*. *coli* strains. Genetic exchange among pathogenic and non-pathogenic strains sharing the same ecological niche has also been demonstrated in other bacterial genera. A comparison between human commensal and pathogenic Neisseria genomes [63], revealed that the former serve as reservoirs of virulence genes and actively engage in horizontal gene transfer with their pathogenic counterparts. Similarly, evolutionary studies of *Streptococcus pneumoniae* and their close commensal relatives have shown that both bacterial lineages presumably evolved from a pathogenic ancestor that lost certain virulence genes during host adaptation to a commensal life style [64]. Horizontal genetic transfer in *E*. *coli* strains can presumably lead to different outcomes including: 1) hybrid DEC strains such as the virulent hybrid EAEC-STEC isolate, *E*. *coli* O104:H4, that caused a large outbreak in Europe in 2011 [65], 2) strains with increased virulence that may cause major epidemiologic shifts in DEC diarrhoea (possibly lt-producing ETEC), and 3) virulent strains that do not belong to any well-defined DEC group, as is likely the case for many non-DEC isolates recovered from patients with diarrhoea.

In summary, our results show that DEC groups are a major cause of moderate to severe diarrhoea in Mexican children. The presence of multiple SVG in these strains, which are more frequent in older children, suggests that the pathogenic capacity of these strains may be conferred by a combination of virulence genes rather than the presence or absence of a single gene. Moreover, when faced with selective immune pressure in a given population, strains will require a repertoire of virulence genes to produce disease.

## Supporting Information

S1 Checklist STROBE checklist. (DOCX)
